# Co-administration of lipopolysaccharide and d-galactosamine induces genotoxicity in mouse liver

**DOI:** 10.1038/s41598-021-81383-5

**Published:** 2021-01-18

**Authors:** Wenjing Dong, Erqun Song, Yang Song

**Affiliations:** Key Laboratory of Luminescence and Real-Time Analytical Chemistry (Southwest University), Ministry of Education, College of Pharmaceutical Sciences, Southwest University, Beibei, Chongqing, 400715 People’s Republic of China

**Keywords:** DNA, DNA

## Abstract

The acute liver injury (ALI) and hepatic fibrosis caused by the co-treatment of lipopolysaccharide (LPS)/d-galactosamine (D-GalN) have been extensively studied. However, whether LPS/D-GalN are genotoxic has been left unknown. In this study, male mice were divided into eight groups with eight animals in each group. For acute challenge of LPS/D-GalN, the mice in each group received a combination of LPS/D-GalN via intraperitoneal injection at the dose of 25 μg/kg/250 mg/kg, 25 μg/kg/500 mg/kg, or 50 μg/kg/500 mg/kg body weight. An additional group for chronic administration of test compounds was conducted by *i.p.* injection of LPS/D-GalN (10 μg/kg/100 mg/kg) every other day for 8 weeks. Saline solution (0.9%) and cyclophosphamide (CTX) (50 mg/kg body weight) given by *i.p.* injection was used as the negative and positive control, respectively. The results of single cell gel electrophoresis (SCGE) assay indicated that acute exposure of the mice to LPS/D-GalN caused severe DNA damage in hepatic cells, but not in the brain, sperm or bone marrow cells, which evidenced the genotoxicity of LPS/D-GalN administrated in combination. Interestingly, the chronic administration of LPS/D-GalN triggered significant genotoxic effects not only in hepatic but also in brain cells, with negative results in sperm and bone marrow cells. Histopathological examination in the liver and brain tissues revealed changes consistent with the SCGE results. The present study indicates genotoxic potential of LPS/D-GalN co-administered in mice, which may serve as an in vivo experimental model for relevant genotoxic study.

## Introduction

Lipopolysaccharide (LPS) is the main endotoxin constituent of the cell wall of gram-negative bacteria, which is important in endotoxic injury^1^. d-galactosamine (D-GalN) may be selectively metabolized by the hepatocytes that are deficient in uridine nucleotides, and it may inhibit macromolecule synthesis in hepatocytes^2^. Liver is the main target organ for LPS, and D-GalN significant worsens the fatal consequence of LPS. Therefore, acute co-injection of LPS/D-GalN is a widely used experimental model for acute liver injury (ALI)^3^, whilst the long term and low-dose treatment of LPS/D-GalN provokes chronic inflammatory responses that resembles hepatic fibrosis^4^.

LPS/D-GalN-caused hepatotoxicity is related to increased expression of pro-inflammatory cytokines, e.g., tumor necrosis factor-α (TNF-α), interleukin-1β (IL-1β) and interleukin-6 (IL-6), along with the generation of reactive oxygen species (ROS) and activates downstream signaling cascade^5,6^. In fact, ROS may act as the initiator of pro-inflammatory cascades^7^. Similarly, environmental pollutants also exhibit their toxicities through ROS-driven inflammatory reaction, especially during their metabolism pathways^8–10^. Beside pro-inflammatory responses, the formed free radicals are readily reactive with DNA, which is strongly implicated in the genotoxic potential of LPS. Previously, a few reports indicated that LPS challenge results in genotoxic effect in different cell lines, including human peripheral blood mononuclear cells, pre-implanting embryonic and uterine cells^11,12^. Since the combination treatment of LPS/D-GalN may amplify radical production, the genotoxic effect of LPS/D-GalN co-administration needs to be investigated.

Here, we hypothesized for a direct link between LPS/D-GalN exposure and ROS-driven genotoxicity. To address our hypothesis, we then determined the genotoxic potential of LPS/D-GalN in mice, by using the alkaline single cell gel electrophoresis (SCGE) assay to evaluate the integrity of genome (DNA breaks). Both acute and chronic treatment of LPS/D-GalN were applied, since they may represent different pathologic patterns.

## Materials and methods

### Chemicals and reagents

LPS (CAS number: 326589-90-6), D-GalN (CAS number: 1772-03-8) and CTX (CAS number: 50-18-0) were purchased from Aladdin Reagent Database Inc. (Shanghai, China). All the other reagents reached the analysis level.

### Animal treatment

Male Kunming mice aged 8 weeks (weight 18–22 g) were obtained from Chongqing Academy of Chinese Materia Medica. The housing and treatment of these animals were approved by the Animal Care Committee of Southwest University and were in accordance with the guidelines of National Institutes of Health guidelines. Mice were contained in cages under 50% humidity, 22 ± 2 °C in a 12 h dark and light cycle. Mice were caged with free access to food and water. Mice were acclimated for 7 days, then were randomly separated into 8 groups, with each experimental group containing eight animals. For the acute co-treatment of LPS/D-GalN, mice were given a single dose of LPS/D-GalN (25 μg/kg + 250 mg/kg, 25 μg/kg + 500 mg/kg, 50 μg/kg + 500 mg/kg body weight) via intraperitoneal (i.p.) injection, and were scarified 6 h after the administration^13^. For the chronic administration, mice were injected *i.p.* with LPS/D-GalN (10 μg/kg + 100 mg/kg) every other day for 8 weeks. Saline solution (0.9% NaCl) and cyclophosphamide (CTX) (50 mg/kg body weight), also given by injected *i.p.*, were chosen as negative and positive control, respectively. At the end of experiments, mice were anesthetized with isoflurane and euthanized with regard for alleviation of suffering. The study was carried out in compliance with the ARRIVE guidelines.

### SCGE assay (comet assay)

After sacrifice of the mice, samples of the liver, brain, testis and bone marrow were extracted for the following experiments. Cells were diluted to about 1 × 10^6^ cells/mL with PBS and refrigerated at 4 °C. One hundred microliters of normal-melting agarose were placed on single surface grinding board at 4 °C for 40 min to get cooled and curdled. The cell suspension (50 µL) and low-melting agarose (50 µL) were mixed and spread on glass slides for 40 min at 4 °C. Finally, the sides were covered with low-melting agarose (100 µL) for 20 min at 4 °C. The slides were immersed in a pre-prepared lysing solution at darkness at 4 °C for 1 h. The slides were next immersed in a cold alkaline solution (pH > 13) for 30 min to precipitate DNA. Thereafter, electrophoresis (25 V, 300 mA) was performed for 30 min. The cells were neutralized by neutralizing fluid (0.4 M Tris, pH 7.5), and stained by using 10 µg/mL ethidium bromide. The comet's image was captured by a reversed fluorescence microscope (OLYMPUS IX71). Next, fifty cells from each of three independent experiments were analyzed with Comet Assay Software Project (CASP) 1.2.2. The tail length (measured from the right edge of the comet head), tail DNA percentage and tail moment (tail length × tail DNA percentage) represent the degree of DNA damage^14^.

### Histopathological examination

Histopathological examination was used to reveal tissue damage. The whole brain and liver of the pretreated mice were obtained, and tissues were soaked in 4% paraformaldehyde and buried by paraffin bag. The fixed brain and liver were cut into sections with thickness of 5 μm. After hematoxylin–eosin (HE) staining, these slides were photographed with a microscope (TE2000, Nikon, Japan).

### Statistical analysis

The results were expressed as means ± standard deviations (S.D). with three independent experiments, and the statistical significance of differences was evaluated by a one-way ANOVA. P < 0.05 was judged as statistically significant.

## Results

**Figure 1:**
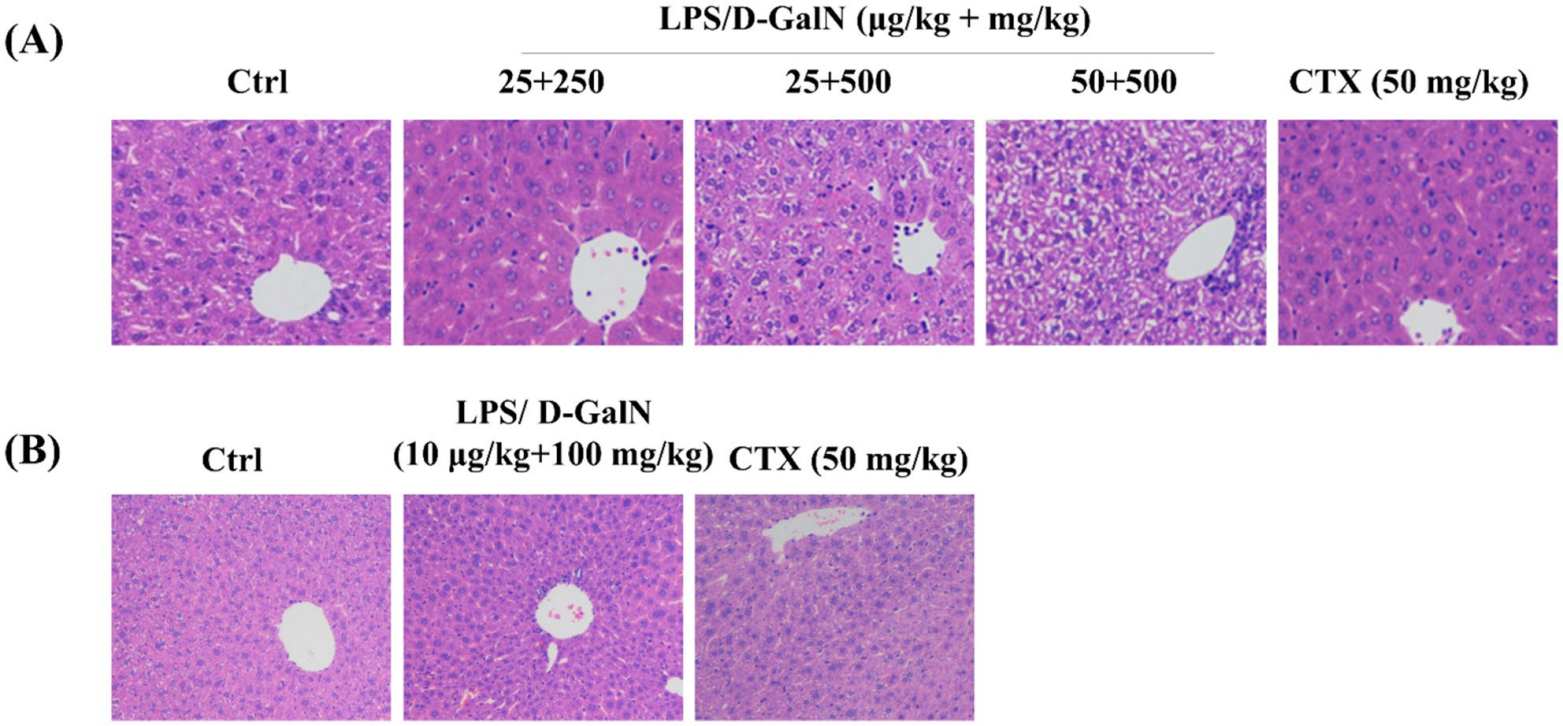
HE staining indicating LPS/D-GalN-induced liver damage. (**A**) Acute treatment. Mice were injected with LPS/D-GalN (25 μg/kg + 250 mg/kg, 25 μg/kg + 500 mg/kg and 50 μg/kg + 500 mg/kg body weight). After 6 h, HE staining was used to show the lesions of the liver in mice. (**B**) Chronic treatment: mice were injected with LPS/D-GalN (10 μg/kg + 100 mg/kg body weight) every other day for 8 weeks. Then HE staining was used to show the lesions of the liver in mice. Saline solution (0.9%) and cyclophosphamide (CTX) (50 mg/kg body weight) were used as negative and positive control, respectively. Original magnification: ×400.

In this study, histopathological examination was used to detect the damage of mice liver. Acute treatment of LPS/D-GalN at the tested doses induced obviously morphological changes, including hepatic sinusoid hyperemia, a loss of normal hepatic architecture and an infiltration of macrophages surrounding the central vein (as shown in Fig. 1A). The effect of chronic treatment of LPS/D-GalN in mice was featured by hepatic fibrogenesis (Fig. 1B), which is in accordance to previous observations by immunohistochemical analysis of mouse liver section^15^. These results suggested successful establishment of ALI and hepatic fibrosis models.

**Figure 2:**
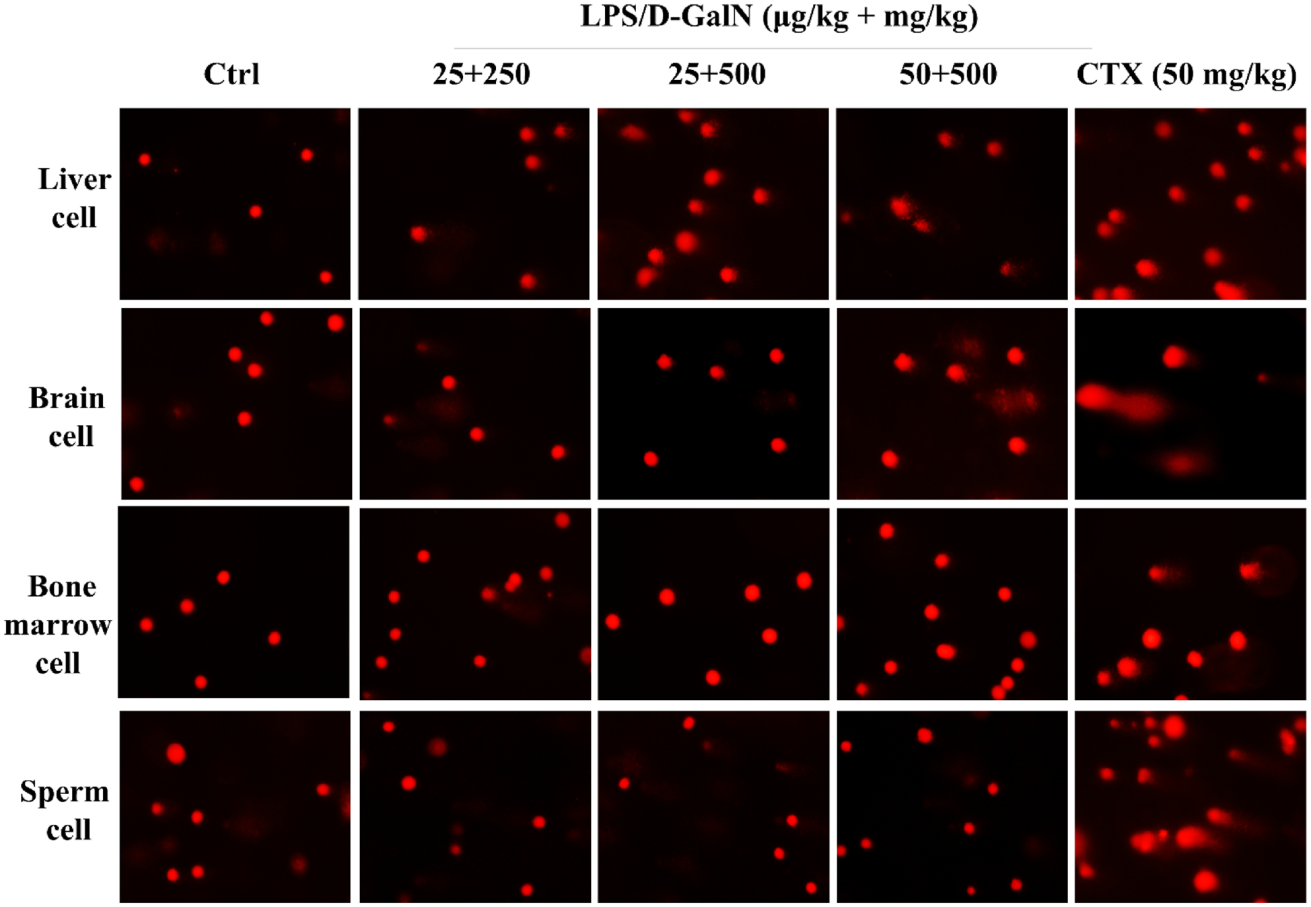
SCGE assay indicating acute LPS/D-GalN exposure induced DNA damage in different cells. Mice were injected intraperitoneally with LPS/D-GalN (25 μg/kg + 250 mg/kg, 25 μg/kg + 500 mg/kg and 50 μg/kg + 500 mg/kg body weight), which were sacrificed after 6 h for tissue sample extraction. Saline solution (0.9%) and cyclophosphamide (CTX) (50 mg/kg body weight) were used as negative and positive control, respectively. SCGE assay was performed using cells from each tissue. The comet's trailing image was captured by a fluorescence microscope (OLYMPUS IX71).

Oxidative DNA damage includes base and sugar lesions, DNA–protein or DNA-DNA crosslinks, single or double-strand breaks, abasic sites and other exocyclic DNA adducts^16^. Herein, we used SCGE assay to score DNA damage occurring in LPS/D-GalN challenged mice. The acute injection of LPS/D-GalN resulted in dose-dependent increases of tail moment, tail DNA (%) and olive tail moment in mice hepatic cells (see Table 1 and Fig. 2).

**Table 1:** DNA damage in cells from the liver, brain, bone marrow and testes (sperm) extracted from mice acutely administered with LPS/D-GalN.

	Tail DNA (%)	Tail moment	Olive tail moment
**Liver cell**			
Ctrl			
0.9% NaCl	3.61 ± 0.51	1.41 ± 0.24	2.17 ± 0.25
LPS/D-GalN			
25 µg/kg + 250 mg/kg	7.19 ± 0.42**	3.66 ± 0.27	4.04 ± 0.37
25 µg/kg + 500 mg/kg	7.80 ± 0.43**	4.46 ± 0.75	4.69 ± 0.59*
50 µg/kg + 500 mg/kg	9.94 ± 0.53***	7.50 ± 0.87***	6.22 ± 0.46***
CTX			
50 mg/kg	13.12 ± 1.37***	11.0 ± 0.27***	8.18 ± 1.12***
**Brain cell**			
Ctrl			
0.9% NaCl	7.66 ± 0.87	3.31 ± 0.51	1.34 ± 0.21
LPS/D-GalN			
25 µg/kg + 250 mg/kg	9.11 ± 0.84	3.78 ± 0.50	0.74 ± 0.12
25 µg/kg + 500 mg/kg	8.89 ± 0.97	4.31 ± 0.68	0.59 ± 0.10
50 µg/kg + 500 mg/kg	6.64 ± 1.10	4.71 ± 1.02	1.79 ± 0.32
CTX			
50 mg/kg	23.20 ± 1.62***	22.59 ± 2.01***	7.69 ± 0.80***
**Bone marrow cell**			
Ctrl			
0.9% NaCl	1.39 ± 0.16	0.32 ± 0.06	0.72 ± 0.11
LPS/D-GalN			
25 µg/kg + 250 mg/kg	2.68 ± 0.29	0.83 ± 0.14	1.30 ± 0.15
25 µg/kg + 500 mg/kg	1.80 ± 0.22	0.40 ± 0.09	0.85 ± 0.11
50 µg/kg + 500 mg/kg	2.22 ± 0.26	1.14 ± 0.21	1.45 ± 0.12
CTX			
50 mg/kg	9.70 ± 1.14***	6.72 ± 1.21***	6.77 ± 0.91***
**Sperm cell**			
Ctrl			
0.9% NaCl	2.76 ± 0.62	0.81 ± 0.28	0.66 ± 0.14
LPS/D-GalN			
25 µg/kg + 250 mg/kg	0.89 ± 0.12	0.24 ± 0.16	0.27 ± 0.09
25 µg/kg + 500 mg/kg	2.10 ± 0.30	0.57 ± 0.12	0.62 ± 0.09
50 µg/kg + 500 mg/kg	2.90 ± 0.26	0.47 ± 0.05	0.84 ± 0.10
CTX			
50 mg/kg	17.38 ± 8.11***	8.20 ± 0.96***	5.1 ± 0.28***

Mice were injected with LPS/D-GalN (25 μg/kg + 250 mg/kg, 25 μg/kg + 500 mg/kg and 50 μg/kg + 500 mg/kg body weight), and after 6 h mice were sacrificed and tissue samples collected for subsequent examination. Saline solution (0.9%) and cyclophosphamide (CTX) (50 mg/kg body weight) were used as negative and positive control, respectively. SCGE assay was performed using cells from each tissue. Data are expressed as means ± S.D.; *P < 0.05, **P < 0.01 and ***P < 0.001, compared with from the control group.

Previous studies illustrated that administration of LPS in mice induced inflammation in the brain^17^, testicles^18^ and bone marrow in mammalians^19^. Now, in this study we investigated whether LPS/D-GalN show similar effects in these targets. Our results showed no significant DNA damage in the brain, testes or bone marrow upon acute administration of LPS/D-GalN (Table 1). CTX was used as the positive control to verify the validity of our experimental results. CTX showed positive genotoxic effects not only in the hepatic cells, but also cells in the other three tissues, which seems consistent with previous studies^20–24^. These data may imply different pharmokinetics and pharmacodynamics between CTX and LPS/D-GalN in our experimental model. Thus, in our acute injection of LPS/D-GalN model, only the major target organ liver shows positive result.

**Figure 3:**
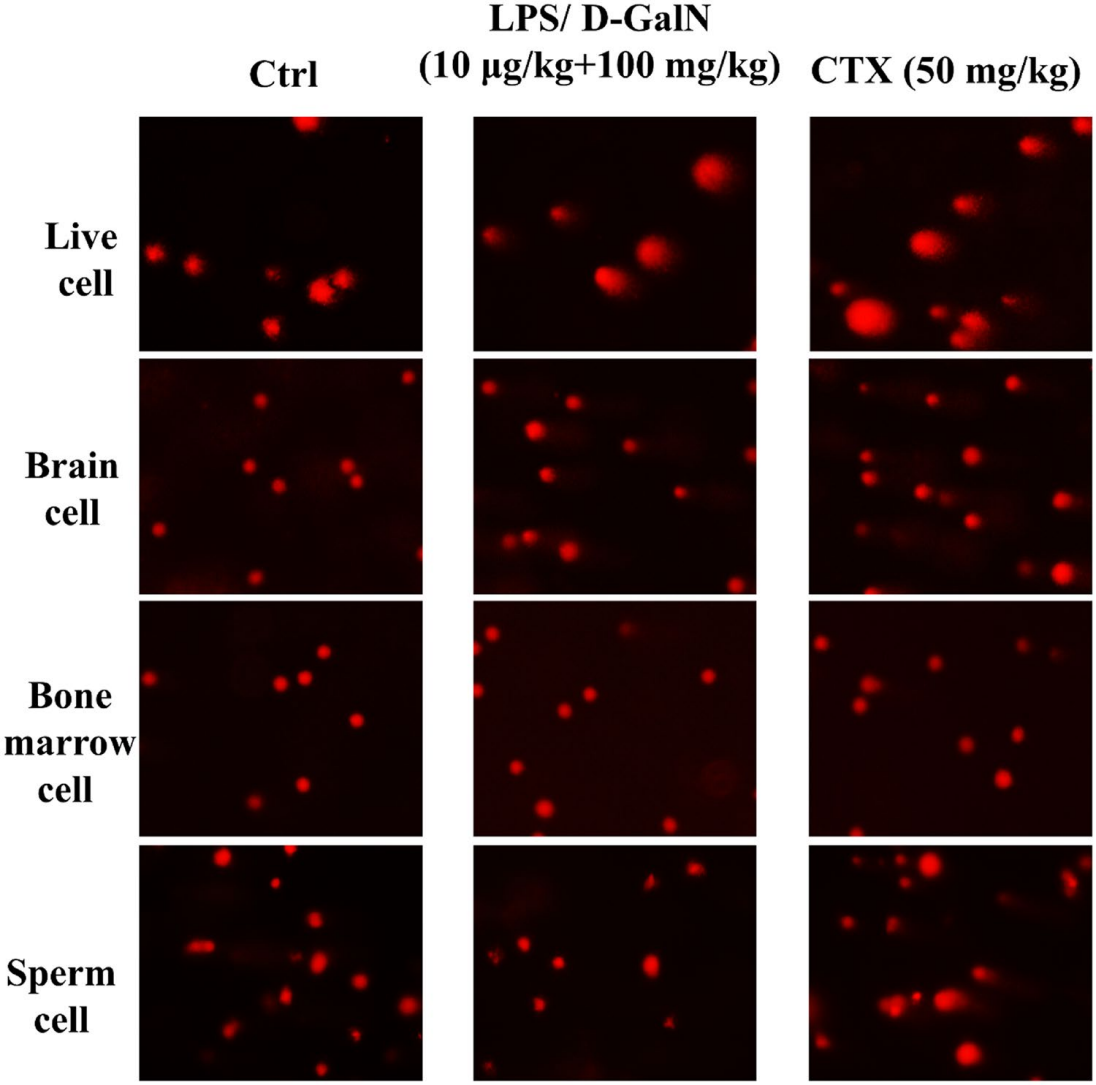
SCGE assay indicating chronic LPS/D-GalN exposure induced DNA damage in different cells. Mice were injected peritoneally with LPS/D-GalN (10 μg/kg + 100 mg/kg body weight) every other day for 8 weeks. Saline solution (0.9%) and cyclophosphamide (CTX) (50 mg/kg body weight) administered in the same conditions as above were used as negative and positive control, respectively. SCGE assay was performed using cells from each tissue. The comet's trailing image was captured by a fluorescence microscope (OLYMPUS IX71).

Although liver is the major target for LPS/D-GalN challenge, the pro-inflammatory cytokines may be released from the target organ which may result in chronic systemic inflammation. It is currently unknown whether chronic LPS/D-GalN challenge induces ROS propagation through liver to other organs. Surprisingly, compared to the negative control, the chronic administration of LPS/D-GalN induced clear DNA migration in the hepatic and brain cells in mice, with statistically significant elevations of tail moment, tail DNA (%) and olive tail moment, as observed in cells from both brain and liver (Table 2 and Fig. 3).

**Table 2:** DNA damage in cells from the liver, brain, bone marrow and testes (sperms) extracted from mice chronic administered with LPS/D-GalN.

	Tail DNA (%)	Tail moment	Olive tail moment
**Liver cell**			
Ctrl			
0.9% NaCl	1.4161 ± 0.90	0.46 ± 0.11	0.25 ± 0.10
LPS/D-GalN			
10 µg/kg + 100 mg/kg	31.8718 ± 9.86***	33.50 ± 3.64***	21.22 ± 8.33***
CTX			
50 mg/kg	23.4090 ± 8.89***	20.16 ± 1.87***	10.45 ± 4.34***
**Brain cell**			
Ctrl			
0.9% NaCl	2.96 ± 0.38	0.42 ± 0.10	0.69 ± 0.10
LPS/D-GalN			
10 µg/kg + 100 mg/kg	38.42 ± 16.52***	36.51 ± 4.93***	20.72 ± 2.48***
CTX			
50 mg/kg	26.76 ± 11.15***	19.90 ± 1.87***	11.12 ± 0.91***
**Bone marrow cell**			
Ctrl			
0.9% NaCl	0.94 ± 0.16	0.04 ± 0.01	0.20 ± 0.02
LPS/D-GalN			
10 µg/kg + 100 mg/kg	1.88 ± 0.08	0.18 ± 0.03	0.44 ± 0.06
CTX			
50 mg/kg	9.08 ± 0.13***	1.58 ± 0.26***	2.62 ± 0.33***
**Sperm cell**			
Ctrl			
0.9% NaCl	3.75 ± 0.59	0.47 ± 0.08	0.94 ± 0.16
LPS/D-GalN			
10 µg/kg + 100 mg/kg	2.68 ± 0.41	0.25 ± 0.05	0.58 ± 0.09
CTX			
50 mg/kg	7.46 ± 0.51**	1.58 ± 0.26**	1.90 ± 0.17**

Mice were peritoneally injected with LPS/D-GalN (10 μg/kg + 100 mg/kg body weight) every other day for 8 weeks. Saline solution (0.9%) and cyclophosphamide (CTX) (50 mg/kg body weight) given in the same mode as described above were used as negative and positive control, respectively. SCGE assay was performed using cells from each tissue. Data are expressed as means ± S.D.; *P < 0.05, **P < 0.01 and ***P < 0.001, compared with the control group.

**Figure 4:**
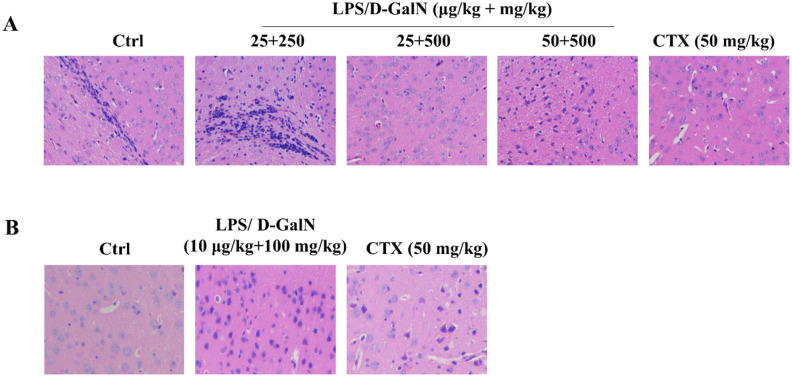
Pathological examination of tissue sections indicating the brain damage. (**A**) Mice were peritoneally injected with LPS/D-GalN (25 μg/kg + 250 mg/kg, 25 μg/kg + 500 mg/kg and 50 μg/kg + 500 mg/kg body weight), 6 h later mice were sacrificed with tissue samples isolated from each mouse. Saline solution (0.9%) and cyclophosphamide (CTX) (50 mg/kg body weight) were used as negative and positive control, respectively. (**B**) Mice were injected with LPS/D-GalN (10 μg/kg + 100 mg/kg body weight), every other day for 8 weeks. Saline solution (0.9%) and cyclophosphamide (CTX) (50 mg/kg body weight) given in the same conditions as above were used as negative and positive control, respectively. Original magnification: ×200.

Histopathological examination of the brain sections from mice chronic administered with LPS/D-GalN showed wrinkled cerebral cortex neurons, which was similar to a previous report^25^. There was no significant change in the cerebral cortex in mice acutely administered with LPS/D-GalN (Fig. 4). However, no DNA damage in bone marrow or sperm cells after chronic LPS/D-GalN administration were observed (Table 2). CTX showed strong genotoxic effect in the cells of the four different types. These results might suggest accumulation of LPS/D-GalN in the brain after long-term administration; however, the exact molecular mechanism needs further investigation.

## Discussion

LPS is known to mimic the pathophysiology of gram-negative bacterial infections, the combined treatment of LPS and D-GalN is a widely acknowledged hepatic injury model^26,27^. The results of this study suggest that co-administration of LPS/D-GalN has strong genotoxic effect in mouse liver, as evidenced by a noteworthy increase in tail DNA (%), tail moment and olive tail moment tail moment in the hepatic cells of the exposed mice. Actually, Zhou et al. observed that LPS/D-GalN administration causes apoptotic DNA fragmentation in mouse liver, an evidence for induction of apoptosis which is different from genotoxicity^28^. Our findings are important because they suggest that the combined administration of LPS/D-GalN may serve as an investigational model for genotoxic study.

Although liver is the major target for LPS insult, LPS-associated damages have been investigated in different organs, for instance, the lungs, heart and kidneys^29–31^. From our present study it can be concluded that acute treatment of the mice with LPS/D-GalN may cause genotoxic consequences only in cells in the liver, whilst chronic administration with LPS/D-GalN may induce genotoxicity in both the liver and brain cells. Neither acute nor chronic treatment of LPS/D-GalN induce significant genotoxic effect in the bone marrow or sperm cells. Noteworthily, the bio-distribution of LPS/D-GalN has not been documented, and the accumulation of LPS/D-GalN in the central nervous system is probably insufficient for eliciting a genotoxic effect in the brain cells of the mice in the acute injection model. In addition, our data also reflect different sensitivity between tissues in response to LPS/D-GalN challenge.

Brain inflammation is associated with many age-related central nervous system diseases^32,33^. Although there is little likelihood that the brain suffers direct endotoxin insult, pro-inflammatory neurodegeneration in the brain can be induced by head trauma^34^, ischemia^35^, and protein aggregates of amyloid β^36^, which are commonly supposed to lead to perpetuated release of neurotoxic cytokines. The chronic but not acute LPS/D-GalN treatment resulted in genotoxicity in the brain cells as observed in this study, implies that although the model is widely used to study liver damage, the brain may not be the primary target.

LPS is not only an endogenous but also an exogenous toxin that generates free radicals, *e.g.*, hydroxyl and nitric oxide radical. Thereafter, the principal mechanism for the toxicity of LPS is known to cause damage to DNA^37^. Continuous high level of ROS in a tissue may result in persistent DNA damage^38^, genome instability^39^, and furthermore cancer initiation and progression^40^. D-GalN co-administration may further amplify ROS production. In this regard, LPS/D-GalN co-administration was previously believed to evoke complications, such as liver sepsis, subsequent to bacterial infections^41^. Here, using SCGE assay, we have demonstrated that LPS/D-GalN challenge induces DNA damage in vivo. Our results are in accordance to those obtained by Verma et al., in which tissue inflammation and genotoxicity in mice occurred following LPS challenge^42^.

In a general perspective, enzymatic antioxidants and non-enzymatic antioxidants play an essential role in protecting cells from ROS insult through the elimination of ROS propagation. Indeed, numerous studies have been conducted for screening anti-inflammatory drugs or compounds in LPS-treated animal models^43,44^. In addition, various antioxidants have been applied in the study of their protective role in LPS/D-GalN-induced toxicity. Abdulazeez et al*.* observed that lycopene is protective against LPS/D-GalN-induced elevation of lipid peroxides, the loss of antioxidative enzyme activities, as well as DNA damage^45^. It is well known that the biologic consequences of LPS challenge include the release of cytokines. However, to the best of our knowledge, there has been no information regarding the use of LPS/D-GalN in a genotoxicity animal model. Besides of the subsequent elucidation of anti-inflammatory parameters, this study might provide information on potential therapeutics for both chronic and acute genotoxic processes. Notably, the major mechanism for LPS/D-GalN induced liver (and brain) damage may belong to an ROS challenge, no matter which endpoints are subsequently involved.

Up to now there have been several models for chemical-induced liver injury as employed in relevant studies of acute/chronic liver injury, including those using acetaminophen, carbon tetrachloride, retrorsine, Fas ligand, and concanavalin A as hepatotoxicants^46,47^. Each of these models has its strengths and weaknesses. In this study, acute and chronic challenge of LPS/D-GalN induce genotoxicity in vivo*,* and the usefulness of this model for relevant genotoxicity studies or as a positive control, is worthy of further investigations.
